# Immunoglobulin G4 disease-related retroperitoneal fibrosis: A series of five cases

**DOI:** 10.4102/sajr.v28i1.2830

**Published:** 2024-05-13

**Authors:** Mohd Ilyas, Shwait Sharma, Vikrant Gupta

**Affiliations:** 1 Department of Radiology, Aster Hospital, Sharjah, United Arab Emirates; 2 Department of Radiology, Government Medical College, Jammu, India

**Keywords:** immunoglobulin G4-related disease, CT, MRI, retroperitoneal fibrosis, aorta, ureter

## Abstract

Immunoglobulin G4 (IgG4)-related disease has the potential to impact any part of the body, including the walls of large- and medium-sized blood vessels and the ureters. While histopathologic examination is currently the standard method for identifying organ involvement and diagnosing IgG4-related disease (IgG4-RD), obtaining biopsy or surgical samples from vessel or ureteral walls is challenging. Given that patients may display only mild symptoms, non-invasive imaging plays a vital role in both diagnosing and managing IgG4-related diseases. Multidetector CT scans are valuable in establishing the primary diagnosis, identifying anatomical landmarks and assessing their relationships. Involvement of the genitourinary organs, such as the ureter, bladder, urethra, and male and female reproductive organs in IgG4-RD, is infrequent when compared to kidney involvement. The imaging findings may include the presence of a localised mass within or surrounding the affected organ or a generalised enlargement of the organ. This report includes cross-sectional images of five cases of IgG4-RD involving large- and medium-sized blood vessels (the aorta and superior mesenteric artery) and the ureters.

**Contribution:**

This case series provides insight into the various imaging appearances of IgG4-related retroperitoneal organ involvement and helps differentiate it radiologically from retroperitoneal fibrosis.

## Introduction

Immunoglobulin G4 (IgG4)-related disease (IgG4-RD) is a condition mediated by the immune system that is increasingly acknowledged in recent years. The pathophysiology includes abnormal interleukin activation which results in fibrosis and becomes evident as a fibrotic mass on imaging and histology. It encompasses a range of disorders that were previously considered unrelated but are now known to possess similar pathological, serological and clinical characteristics. Among the shared features are the presence of IgG4-positive plasma cells and lymphocytes infiltrating the affected tissues, accompanied by fibrosis. The diagnostic factors involve a radiological observation described as (1) low attenuation thickening or soft tissue masses surrounding the aorta and its major branches, (2) soft tissue masses located in the wall of the renal pelvis and/or around the ureter or (3) soft tissue masses present in the pelvis and paravertebral region. The newly proposed diagnostic criteria specific to individual organs are that periaortitis or periarteritis and retroperitoneal fibrosis can be identified as IgG4-RD based on histological findings, irrespective of the levels of IgG4 in the bloodstream.^1,2^

## Case series

### Case 1

**FIGURE 1:**
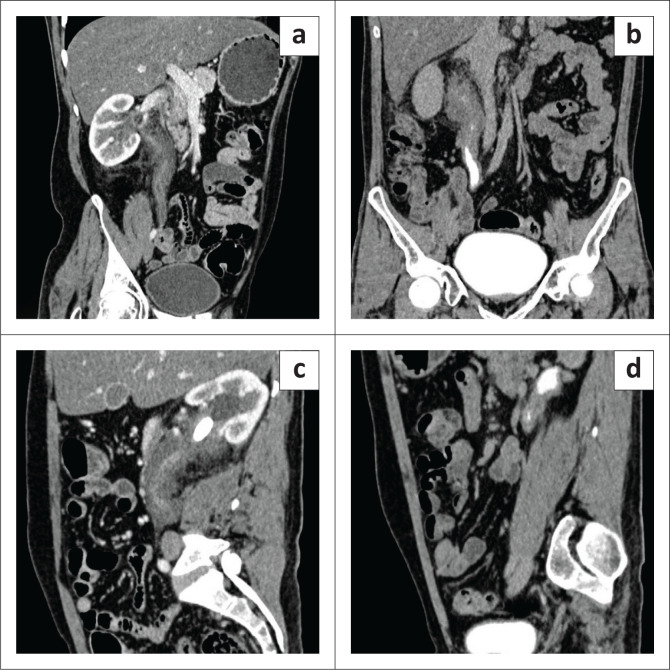
Coronal reformatted (a–b) and sagittal (c–d) sections of the contrast CT abdomen of case 1 show (a–c) a circumferential cuff of non-enhancing soft tissue mass encircling the right upper ureter and renal pelvis, extending for a length of approximately 12 cm along the upper ureter; (d) cuff of soft tissue mass involving the left upper ureter causing luminal effacement.

A 35-year-old male who was recently diagnosed with bilateral nephrolithiasis was referred for CT urography. The images obtained revealed multiple, hyperdense calculi in the right renal pelvis, the largest measuring 28 mm × 21 mm with a mean attenuation value of 1230HU. There was associated marked peripelvic fat stranding and a cuff of non-enhancing soft tissue encircling the right upper ureter and renal pelvis, measuring 33 mm × 28 mm (Anteroposterior [AP] × transverse) extending for a length of approximately 12 cm along the upper ureter (Figure 1a–c). The left kidney revealed a 7 mm × 8 mm hyperdense calculus with a mean attenuation value of 780HU in the left upper ureter with a similar non-enhancing soft tissue mass cuffing the left renal pelvis and upper ureter for a length of 7 cm – 8 cm, maximum AP and transverse diameters of 23 mm × 12 mm, respectively (Figure 1d).

CT-guided fine needle aspiration and biopsy revealed a few focal areas of stromal fibrosis without any evidence of malignancy. Initial blood serology was unremarkable. An autoimmune profile was requested, including serum IgG4 levels, which revealed markedly elevated serum IgG4 levels (> 300 mg/dL). The patient underwent bilateral double-J (DJ) stent insertions and was commenced on corticosteroid therapy for IgG4-RD.

### Case 2

**FIGURE 2:**
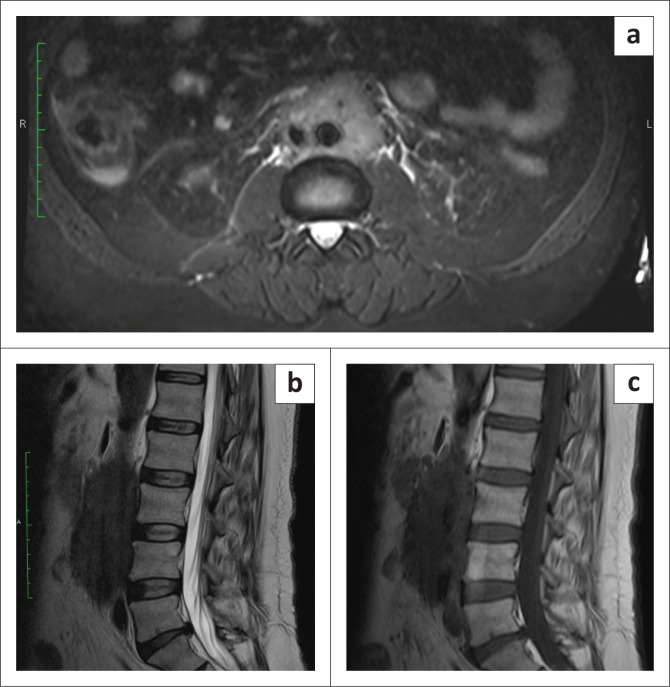
Axial (a) and sagittal (b–c) MR images of the lumbar spine of case 2 show a short tau inversion recovery (STIR) hyperintense (a) and T1-weighted and T2-weighted hypointense (b–c) circumferential mass encasing the abdominal aorta and IVC.

**FIGURE 3:**
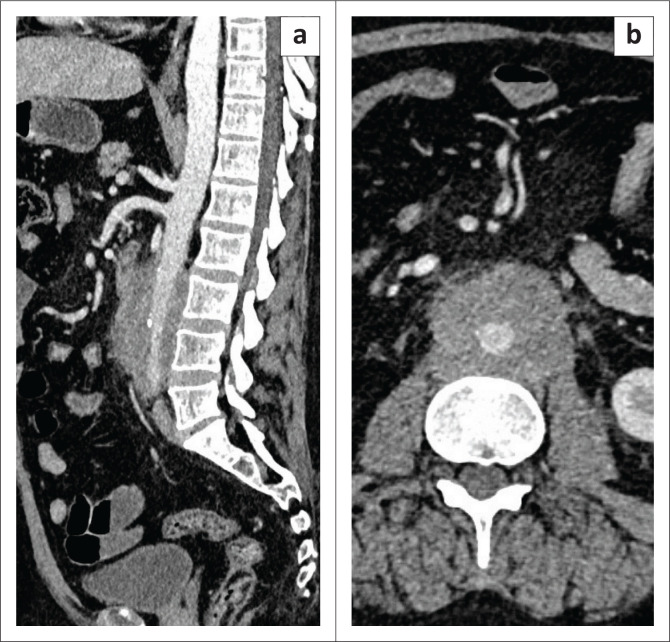
Sagittal reformatted (a) and axial (b) sections of the contrast-enhanced CT abdomen of case 2 demonstrate a fibrotic soft tissue cuff encasing the abdominal aorta with no definite luminal effacement of the aorta.

A 53-year-old male presented with chronic low back ache and was referred for MRI of the lumbosacral spine. Imaging revealed a T1-weighted (T1-W) and T2-weighted (T2-W) hypointense mass surrounding the abdominal aorta, hyperintense in signal on short tau inversion recovery (STIR) images (Figure 2a–c). Initially thought to be a nodal mass, contrast-enhanced CT (CECT) was advised. Abdominal CECT revealed a long segment of circumferential, hypodense, mildly enhancing soft tissue mass with an infiltrative and spiculated appearance in the retroperitoneum, completely encasing the infrarenal aorta and inferior vena cava (IVC), extending along the proximal portions of the common iliac arteries without ureteric involvement (Figure 3a and 3b). The patient was extensively evaluated for autoimmune disorders and retroperitoneal fibrosis and serum IgG4 levels were raised at 235 mg/dL. Following the initiation of corticosteroid therapy, the symptoms improved over time and the patient is currently under follow-up.

### Case 3

**FIGURE 4:**
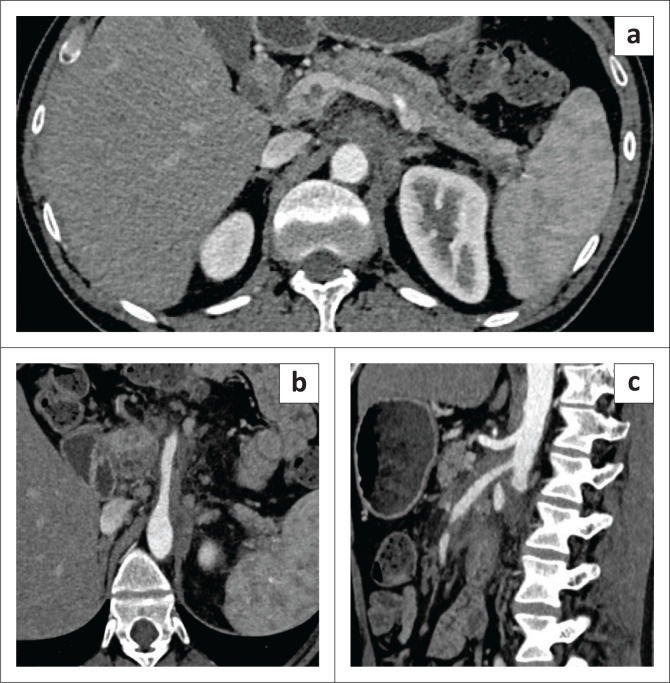
Axial (a–b) and sagittal (c) sections of the CT abdomen of case 3 reveal features of chronic pancreatitis (a), soft tissue cuffing of the long segment of the superior mesenteric artery (b–c).

A 38-year-old male under evaluation for chronic pancreatitis was referred for CECT of the abdomen. The study revealed a mild reduction in the pancreatic bulk and an irregularly dilated main pancreatic duct (4.5 mm). It also revealed a cuff of non-enhancing soft tissue mass encircling the proximal 6.5 cm length of the superior mesentery artery with a maximal thickness of 10 mm, extending into the peripancreatic and/or pancreaticoduodenal groove (Figure 4a–c). The patient was evaluated for autoimmune disorders, but all tests were negative. The serum IgG4 levels were marginally raised (145 mg/dL). A diagnosis of IgG4-RD of the pancreas and superior mesenteric artery was made. The patient was commenced on steroid therapy and the symptoms gradually subsided.

### Case 4

**FIGURE 5:**
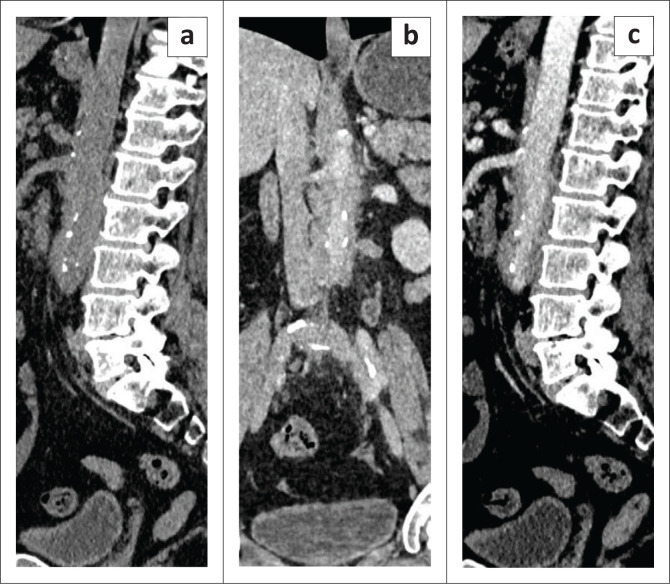
Sagittal (a and c) and coronal (b) sections of the CT abdomen indicate atheromatous calcific plaques on unenhanced CT (a) involving the aorta, with a soft tissue cuff involving the infrarenal abdominal aorta on contrast-enhanced CT (b–c).

A 62-year-old female was referred for CECT of the abdomen for abdominal pain and unexplained weight loss. The study revealed a long circumferential cuff of soft tissue thickening involving the abdominal aorta starting at the level of the renal artery origin and extending for a length of 7.9 cm to the aortic bifurcation with a maximum single wall thickness of 14 mm. No other abnormality was noted at CT imaging (Figure 5a–c). Extensive blood serology for autoimmune vascular disorders revealed elevated IgG4 levels and erythrocyte sedimentation rate. The patient was started on therapy for IgG4-RD and symptoms improved drastically.

### Case 5

**FIGURE 6:**
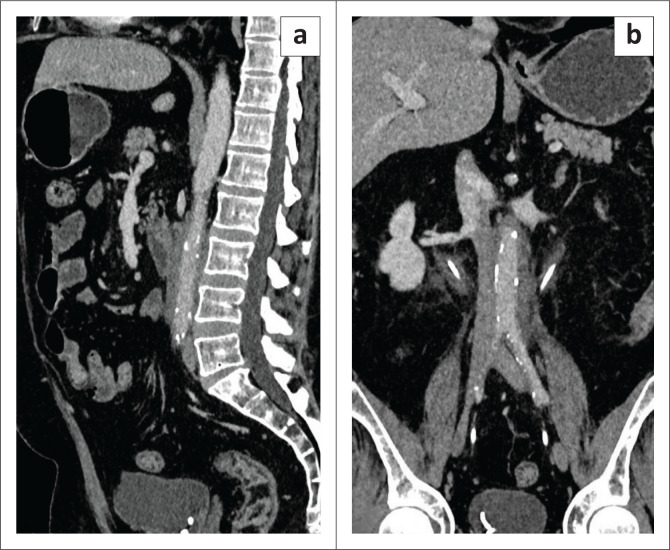
Sagittal (a) and coronal (b) contrast-enhanced CT images show a soft tissue cuff along the abdominal aorta (a–b) and along both upper ureters (b) with ureteric stents in-situ (b).

A 58-year-old male who had undergone bilateral DJ stenting for non-calculous ureteric obstruction was referred for CECT abdomen to evaluate for the cause of the obstruction. Initially, the patient had presented with bilateral renal colic with bilateral hydronephrosis on ultrasonography and no evidence of renal or ureteric calculi. The patient did not undergo CECT at that time due to deranged renal function. The patient subsequently underwent DJ stenting, after which the CECT was performed as the renal function tests normalised. The CT images revealed a circumferential cuff of non-enhancing soft tissue surrounding the abdominal aorta and both upper ureters with periureteric fat stranding causing luminal narrowing of the ureters and aorta (Figure 6a and 6b). A presumptive diagnosis of retroperitoneal fibrosis was made and the patient was evaluated for autoimmune pathology which revealed markedly elevated IgG4 levels (275 mg/dL). The patient started steroid therapy and is under follow-up.

## Discussion

Immunoglobulin G4-related disease can affect nearly all organ systems, with notable affinity for the pancreas, biliary tree, salivary glands, orbits, lacrimal glands, lungs, kidneys, aorta, retroperitoneum, meninges and thyroid gland. Among the genitourinary organs, the kidneys are most commonly involved in IgG4-RD, while inclusion of other organs such as the ureter, bladder and prostate is infrequent.^3^ It is more common in middle-aged males, those with pre-existing autoimmune disorders and allergic or atopic disorders.

Diagnosing IgG4-RD involves a comprehensive assessment combining clinical, serologic, radiologic and histopathologic information.^4^ No single finding can definitively confirm the diagnosis. Cross-sectional imaging, with ultrasound, CT or MRI, plays a crucial role in both diagnosis and management. In cases of hydronephrosis and impaired renal function, retrograde pyelography may be conducted to assess the ureters and collecting systems.^5^ However, imaging results are typically non-specific and variable, especially in the kidneys and lungs, making it challenging to reliably differentiate IgG4-RD from other conditions.^2^ An exception to this challenge is when the pancreatic region is under examination. Distinctive imaging features of autoimmune pancreatitis, such as the pancreas displaying sausage-like enlargement and a peripancreatic halo, can strongly indicate IgG4-RD when identified within the appropriate clinical context. This context includes (1) mild abdominal symptoms, typically without acute pancreatitis attacks, (2) occasional episodes of obstructive jaundice, (3) elevated serum gamma globulin, IgG and/or IgG4 concentrations and (4) sporadic involvement of other organs.^6^ The identification of a peripancreatic halo, indicative of a fibroinflammatory process extending into the surrounding adipose tissue, serves as a valuable imaging finding for diagnosing IgG4-RD and distinguishing it from pancreatic cancer or other forms of pancreatitis.^7^

In IgG4-RD, the urinary tract is most frequently affected.^2^ Kidney involvement is observed in approximately 25%–33% of individuals with IgG4-RD autoimmune pancreatitis, and it can manifest independently, without concurrent involvement of other organs. In the kidney, it can result in various changes in the renal pelvis and renal sinus. IgG4-RD can cause the walls of the renal pelvis to become thicker throughout, resulting in a broader appearance. The inner surface of the renal pelvis may appear smooth without any irregularities or abnormalities. In some cases, a soft tissue nodule may be present within the renal sinus, which is the cavity in the kidney that connects to the renal pelvis. It is common for IgG4-RD of the kidney to affect both the kidneys. However, the absence of bilateral involvement does not completely exclude the possibility of IgG4-RD; unilateral involvement can still be seen in some cases.

Involvement of the ureter in IgG4-RD is rare, and there have been limited reports. Ureteral IgG4-RD has been previously referred to as ‘inflammatory pseudotumour’ or ‘idiopathic segmental ureteritis’. Typically, thickening of the ureteral wall occurs as an extension from the thickened renal pelvis wall in IgG4-RD of the kidney. However, there have been cases where segmental ureteral wall thickening without a concurrent renal pelvis lesion has been observed, leading to concentric thickening, hydronephrosis and hydroureter. Another manifestation of ureteral IgG4-RD is periureteral fibrosis, where disease epicentres are located in the periureteric adipose tissue.^8^ Ureteral IgG4-RD can be categorised into three types based on macroscopic characteristics: polypoid mass-forming lesions, segmental thickening of the ureteral wall and periureteral fibrosis. The presence of obstructive hydroureteronephrosis is commonly associated due to mass effect, irrespective of the site of ureteric involvement.^9^

Compared to renal involvement, IgG4-RD affecting the urinary bladder is uncommon. Isolated cases of bladder IgG4-RD have been documented in a few reports.^10,11^ The bladder manifestations of IgG4-RD include an intravesical polypoid mass resembling bladder cancer, or a subepithelial tumour, such as a leiomyoma. A specific case report detailed IgG4-RD in the bladder, where a well-defined polypoid mass emerged from the bladder wall and exhibited low signal intensity on T2-W MRI. Confirmation of bladder IgG4-RD was achieved through transurethral resection of the mass, revealing its confinement to the subepithelial layer of the bladder.^11^ Currently, there is limited available information regarding the imaging characteristics of IgG4-RD affecting the prostate gland. Only a few case reports have briefly described the CT findings, noting diffuse enlargement of the prostate gland with uniform low attenuation.^12^

IgG4-related aortitis can present with non-specific and vague clinical symptoms, often leading to its initial underdiagnosis, especially in patients without retroperitoneal fibrosis or hydronephrosis. When evaluating IgG4-related cardiovascular disease (CVD) using CECT scans, certain features can be observed. IgG4-related CVD can cause a widespread thickening of the arterial walls, exceeding 2 mm in thickness. The thickened arterial walls typically display a uniform enhancement pattern after contrast administration, indicating increased blood flow to the affected area. In some cases, it can lead to the development of masses within the arteries. These masses may not cause significant narrowing of the blood vessels and can have irregular margins. It may or may not be accompanied by aortic dilatation.^13^ It is important to consider IgG4-related aortitis in the differential diagnosis of patients presenting with these imaging findings, even when the clinical presentation is non-specific and there is an absence of retroperitoneal fibrosis or hydronephrosis. The one differential of retroperitoneal lymphoma can be excluded on the basis of the fact that there will be no mass effect in retroperitoneal fibrosis or IgG4-related disease.

The comprehensive criteria for diagnosing IgG4-RD in routine clinical practice include, as proposed by Inoue et al. and Umehara et al., (1) the presence of characteristic diffuse or localised swelling, or the identification of masses in one or more organs during clinical examination, (2) elevated levels of serum IgG4 concentrations (≥ 135 mg/dL) and (3) histopathologic findings indicating marked lymphoplasmacytic infiltration, storiform fibrosis and infiltration of IgG4-positive plasma cells into organs. A definitive diagnosis of IgG4-related disease is established when all three criteria are met, considered probable when the first and third criteria are fulfilled, and deemed possible when the first and second criteria are satisfied.^14,15^

The key features of CT and/or PET-CT assessment for IgG4-related CVD are outlined as follows: (1) CECT is recommended for a comprehensive evaluation of vessels throughout the body. Optimal assessment of aortitis is achieved in the delayed phase rather than the early phase. (2) For subsequent examinations, utilise CT for comparative analysis to gauge changes in vessel size, including outer and luminal diameters and wall thickness. (3) When assessing disease activity, focus on evaluating the vessel’s maximum standardised uptake value (SUVmax) or the target-to-background ratio, rather than the blood pool SUVmax. (4) For coronary artery evaluation, opt for ECG-gated coronary CT angiography over non-ECG-gated CT. Utilise transaxial and reformatted images, including curved planar reconstruction and images in the vessel short-axis plane. (5) During follow-up examinations, ensure acquisition in the same imaging plane for consistent and reproducible comparisons of vessel size.^1^

The radiologic diagnostic factor involves observation of (1) hypertrophic thickening or the presence of low-attenuating soft tissue masses surrounding the aorta and its major branches, (2) the existence of soft tissue masses around the renal pelvis wall and/or along the ureter or (3) the occurrence of soft tissue masses in the pelvic and paravertebral regions.^16^

The various subtypes and secondary forms of retroperitoneal fibrosis, which include perianeurysmal and non-aneurysmal fibrosis when it develops around aorta, should be considered among the differential diagnosis when the aorta is predominantly involved. Other spectra of retroperitoneal fibrosis to be considered while dealing with such cases include periureteritis fibrosa, periureteritis plastica, chronic periureteritis, sclerosing retroperitoneal granuloma and fibrous retroperitonitis. Once a diagnosis of IgG4-related disease is made, it is important to perform whole-body imaging with either CT or PET-CT to determine the extent of the disease.

## Conclusion

IgG4-RD is a systemic condition that has been recognised relatively recently. It causes the infiltration of organs by fibroinflammatory tissue and can occasionally resemble other inflammatory or neoplastic processes. Familiarity with this disorder is crucial for radiologists, as patients typically exhibit a significant response to corticosteroid therapy. Timely recognition of the condition can assist in avoiding diagnostic delays and unnecessary invasive procedures.

## References

[1] Oyama-Manabe N, Yabusaki S, Manabe O, Kato F, Kanno-Okada H, Kudo K. IgG4-related cardiovascular disease from the Aorta to the Coronary Arteries: Multidetector CT and PET/CT. Radiographics. 2018;38(7):1934–1948. 10.1148/rg.2018180049 30289734

[2] Oh JW, Rha SE, Choi MH, Oh SN, Youn SY, Choi JI. Immunoglobulin G4-related disease of the genitourinary system: Spectrum of imaging findings and clinical-pathologic features. Radiographics. 2020;40(5):1265–1283. 10.1148/rg.2020200043 32870766

[3] Yamamoto M, Takahashi H. IgG4-related disease in organs other than the hepatobiliary-pancreatic system. Semin Liver Dis. 2016;36(3):274–282. 10.1055/s-0036-1584317 27466796

[4] Kamisawa T, Zen Y, Pillai S, Stone JH. IgG4-related disease. Lancet. 2015;385(9976):1460–1471. 10.1016/S0140-6736(14)60720-0 25481618

[5] Geoghegan T, Byrne AT, Benfayed W, McAuley G, Torreggiani WC. Imaging and intervention of retroperitoneal fibrosis. Australas Radiol. 2007;51(1):26–34. 10.1111/j.1440-1673.2006.01654.x 17217486

[6] Okazaki K. Autoimmune pancreatitis and IgG4-related disease: The Storiform discovery to treatment. Dig Dis Sci. 2019;64(9):2385–2394. 10.1007/s10620-019-05746-9 31363956

[7] Zaheer A, Singh VK, Akshintala VS, et al. Differentiating autoimmune pancreatitis from pancreatic adenocarcinoma using dual-phase computed tomography. J Comput Assist Tomogr. 2014;38(1):146–152. 10.1097/RCT.0b013e3182a9a431 24424563 PMC4394855

[8] Martínez-de-Alegría A, Baleato-González S, García-Figueiras R, et al. IgG4-related disease from head to toe. Radiographics. 2015;35(7):2007–2025. 10.1148/rg.357150066 26473450

[9] Kim SA, Lee SR, Huh J, Shen SS, Ro JY. IgG4-associated inflammatory pseudotumor of ureter: Clinicopathologic and immunohistochemical study of 3 cases. Hum Pathol. 2011;42(8):1178–1184. 10.1016/j.humpath.2010.03.011 21334715

[10] Montironi R, Scarpelli M, Cheng L, et al. Immunoglobulin G4-related disease in genitourinary organs: An emerging fibroinflammatory entity often misdiagnosed preoperatively as cancer. Eur Urol. 2013;64(6):865–872 [Published correction appears in Eur Urol 2015;67(4):e85. 10.1016/j.eururo.2012.11.056 23266239

[11] Park S, Ro JY, Lee DH, Choi SY, Koo H. Immunoglobulin G4-associated inflammatory pseudotumor of urinary bladder: A case report. Ann Diagn Pathol. 2013;17(6): 540–543. 10.1016/j.anndiagpath.2013.01.004 23434261

[12] Kufukihara R, Niwa N, Mizuno R, et al. Immunoglobulin G4-related disease arising from the bladder wall. Urol Int. 2019;103(4):488–490. 10.1159/000495570 30544121

[13] Buijs J, Maillette de Buy Wenniger L, Van Leenders G, et al. Immunoglobulin G4-related prostatitis: A case-control study focusing on clinical and pathologic characteristics. Urology. 2014;83(3):521–526. 10.1016/j.urology.2013.10.052 24581512

[14] Inoue D, Zen Y, Abo H, et al. Immunoglobulin G4-related periaortitis and periarteritis: CT findings in 17 patients. Radiology. 2011;261(2):625–633. 10.1148/radiol.11102250 21803920

[15] Umehara H, Okazaki K, Masaki Y, et al. Comprehensive diagnostic criteria for IgG4-related disease (IgG4-RD), 2011. Mod Rheumatol. 2012;22(1):21–30. 10.3109/s10165-011-0571-z 22218969

[16] Mizushima I, Kasashima S, Fujinaga Y, Kawano M, Ishizaka N. IgG4-related periaortitis/periarteritis: An under-recognized condition that is potentially life-threatening. Mod Rheumatol. 2019;29(2):240–250. 10.1080/14397595.2018.1546367 30474460

